# Understanding Engagement Strategies in Digital Interventions for Mental Health Promotion: Scoping Review

**DOI:** 10.2196/30000

**Published:** 2021-12-20

**Authors:** Maham Saleem, Lisa Kühne, Karina Karolina De Santis, Lara Christianson, Tilman Brand, Heide Busse

**Affiliations:** 1 Department of Prevention and Evaluation Leibniz Institute for Prevention Research and Epidemiology - BIPS Bremen Germany; 2 Leibniz Science Campus Digital Public Health Bremen Germany; 3 Department of Administration Leibniz Institute for Prevention Research and Epidemiology - BIPS Bremen Germany

**Keywords:** digital interventions, mental health promotion, engagement, scoping review, mobile phone

## Abstract

**Background:**

Digital interventions offer a solution to address the high demand for mental health promotion, especially when facing physical contact restrictions or lacking accessibility. Engagement with digital interventions is critical for their effectiveness; however, retaining users’ engagement throughout the intervention is challenging. It remains unclear what strategies facilitate engagement with digital interventions that target mental health promotion.

**Objective:**

Our aim is to conduct a scoping review to investigate user engagement strategies and methods to evaluate engagement with digital interventions that target mental health promotion in adults.

**Methods:**

This scoping review adheres to the PRISMA (Preferred Reporting Items for Systematic Reviews and Meta-Analyses) guidelines for scoping reviews. The search was conducted in 7 electronic databases from inception to April 2020. The inclusion criteria for studies were as follows: adult (aged ≥18 years) users of digital interventions for mental health promotion from the general population; any digital intervention for mental health promotion; and user engagement strategies described in the intervention design. We extracted the following data items: study characteristics, digital intervention (type and engagement strategy), evaluation of engagement strategy (method and result specifying whether the strategy was effective at facilitating engagement), and features of engagement (extent of use and subjective experience of users).

**Results:**

A total of 2766 studies were identified, of which 16 (0.58%) met the inclusion criteria. The 16 studies included randomized controlled trials (6/16, 37%), studies analyzing process data (5/16, 31%), observational studies (3/16, 19%), and qualitative studies (2/16, 13%). The digital interventions for mental health promotion were either web based (12/16, 75%) or mobile app based (4/16, 25%). The engagement strategies included personalized feedback about intervention content or users’ mental health status; guidance regarding content and progress through e-coaching; social forums, and interactivity with peers; content gamification; reminders; and flexibility and ease of use. These engagement strategies were deemed effective based on qualitative user feedback or responses on questionnaires or tools (4/16, 25%), usability data (5/16, 31%), or both (7/16, 44%). Most studies identified personalized support in the form of e-coaching, peer support through a social platform, personalized feedback, or joint videoconference sessions as an engaging feature.

**Conclusions:**

Personalized support during the intervention, access to social support, and personalized feedback seem to promote engagement with digital interventions for mental health promotion. These findings need to be interpreted with caution because the included studies were heterogeneous, had small sample sizes, and typically did not address engagement as the primary outcome. Despite the importance of user engagement for the effectiveness of digital interventions, this field has not yet received much attention. Further research is needed on the effectiveness of different strategies required to facilitate user engagement in digital interventions for mental health promotion.

## Introduction

### Background

Mental health promotion and well-being is a global public health challenge because of the high prevalence of mental disorders [1]. Mental health disorders are among the leading causes of global disability-adjusted life years (DALYs), with depressive disorders responsible for 1.8% of the DALYs and anxiety responsible for 1.1% of the DALYs [2]. As such, mental health disorders carry high costs not only for individuals, but also for families, communities, and societies [3]. In the European Union, the costs of mental disorders are estimated as being more than €600 billion (US $694 billion), which represents more than 4% of the gross domestic product across the European Union [3].

Mental health disorders have increased over time globally [2], highlighting the need for the prevention of mental disorders and promotion of mental well-being and mental health of the general population.

To face the challenge of the increasing burden of mental disorders and to address the demand for mental health promotion, technological approaches provide a solution [1]. Digital interventions offer the potential to overcome availability and accessibility barriers, including geographical location and time [1,4,5]. Furthermore, digital interventions for mental health are accessible to internet users who own PCs or mobile devices. Anonymous use is desired to bypass barriers because of the stigma of seeking help for mental health concerns [4,6,7]. Thus, digital interventions may reach different target groups compared with local mental health services [6].

Digital interventions for mental health are defined as interventions that are delivered through a digital platform such as the web [1,4,8], smartphone apps [4,6], SMS text messaging (on any platform) [1], and virtual reality [1,4] and target the prevention or treatment of mental health disorders. These interventions mostly implement techniques related to cognitive behavioral therapy or positive psychology [7] and, in the context of mental health, have been applied in healthy [4,8] and clinical samples [4,6,7]. The effectiveness of such interventions has been addressed by a number of systematic reviews. For example, Lattie et al [4] investigated digital interventions for college students who were either healthy or showed symptoms of psychological distress, depression, or anxiety. The authors found that some interventions, regardless of the type of digital intervention, were effective in improving mental health outcomes, including depression, anxiety, and psychological well-being in general. Furthermore, a systematic review by Weisel et al [6] examined mobile apps for adults with heightened symptom severity of several mental health disorders. Indeed, some interventions such as apps delivering cognitive behavioral therapy were found to be effective in reducing symptoms of depression but not effective in reducing symptoms of anxiety [6]. Overall, the systematic reviews suggest that an important function of digital interventions is to not only address existing clinical symptoms, but also to target the promotion of mental health; in general, enhance mental health promotion. Thus, this scoping review focuses on the application of digital interventions in studies with nonclinical samples for mental health promotion.

Engagement in digital technologies is critical for their effectiveness; however, retaining users’ engagement in digital interventions is challenging [9]. Digital interventions, in general, are prone to attrition because of their self-help and unguided nature [5]. For example, the systematic review by Lattie et al [4] revealed that many digital interventions that targeted the promotion of mental health in college students were effective, but attrition rates (ie, proportion of participants dropping out from the intervention) were high in the investigated trials. In some trials, most of the participants adhered to the first module but did not complete the subsequent modules [4]. Despite some evidence for the effectiveness and benefits of digital interventions for mental health promotion, problems are further encountered in translating the results from research studies into real-life settings [9]. Attrition is frequently reported in real-life settings when using digital interventions for general health and well-being [9] as well as prevention and treatment for specific conditions such as depression [7]. These findings highlight the need to develop strategies to effectively engage users with digital interventions for mental health promotion.

Engagement with digital interventions can be defined as “(1) the extent (e.g. amount, frequency, duration, depth) of usage and (2) a subjective experience characterized by attention, interest, and affect” [10]. The features frequency, duration, and amount refer to temporal use, with “amount” being defined as “total length of each intervention contact.” “Depth” is defined as a “variety of content used” [10]. Accordingly, engagement is described as a multidimensional construct in which users experience sustained behavioral aspects of engagement.

### Objective

A synthesis of evidence on engagement strategies is required for digital interventions that address mental health promotion. Our aim is to collate such evidence using a scoping review approach to obtain a broad understanding of how user engagement is explored, measured, and evaluated in the context of digital interventions for mental health promotion. The research questions are as follows:

1. What strategies are applied to improve user engagement with digital interventions for mental health promotion?
2. What type of strategies result in better engagement with digital interventions for mental health promotion, and how is this improvement in engagement measured?

## Methods

### Methodological Details

This scoping review followed the Joanna Briggs Institute Scoping Review Methodology [11] and is reported based on the Preferred Reporting Items for Systematic Reviews and Meta-Analyses Extension for Scoping Reviews (PRISMA-ScR) guidelines [12]. Additional methodological details are reported in the multimedia appendices. The completed PRISMA-ScR checklist is shown in Multimedia Appendix 1.

### Protocol and Registration

The protocol for this review was prospectively registered at the Open Science Foundation registries [13].

### Eligibility Criteria

Primary studies with any design were eligible for inclusion. The studies had to fulfill the following Population, Intervention, Control, and Outcomes characteristics:

Population: Any users of digital interventions for mental health promotion aged ≥18 years from the general population not in a clinical setting.

Intervention: Digital intervention for mental health promotion.

Control: Any comparator, such as another intervention type, or no comparator;

Outcomes:

1. Any user engagement strategy used in the design of digital interventions for mental health promotion.
2. Effectiveness of engagement strategies assessed and evaluated after the intervention.

The exclusion criteria were as follows:

1. Studies without primary data, including reviews, commentaries, letters to the editor, and study protocols.
2. Studies with clinical samples or specific subpopulations, for example, high-risk groups.
3. Studies with digital interventions for mental health treatment or health-related fields other than mental health.
4. Studies that did not report or recommend engagement strategies in the intervention design.

### Information Sources

The information sources were the following electronic databases:

1. MEDLINE through Ovid
2. CINAHL through EBSCO
3. The Social Science Citation Index through Web of Science
4. The Science Citation Index through Web of Science
5. The Emerging Sources Citation Index through Web of Science
6. PsycINFO through Ovid
7. The Cochrane Central Register of Controlled Trials and the Cochrane Database of Systematic Reviews through the Cochrane Library

### Search Strategy

The search strategy was developed by the team assisted by an experienced information specialist who subsequently conducted the search. Databases were searched from inception to April 2020, with no language limits applied. The search results for each database are presented in Multimedia Appendix 2. The search structure combined appropriate keywords and controlled vocabulary terms for 3 concepts: digital health interventions, engagement, and mental health. The search syntax for MEDLINE is presented in Multimedia Appendix 3.

### Selection of Sources of Evidence

All results were exported to EndNote (Clarivate) reference management software for deduplication and then imported to Covidence (Veritas Health Innovation Ltd) systematic review management software for title, abstract, and full-text screening. In all, 2 authors (MS and LK) independently selected studies based on title or abstract, and any inconsistencies were resolved by consensus during discussion. A list of articles included and excluded for full-text screening are presented in Multimedia Appendix 4.

### Data-Charting Process

A data-charting form was developed and calibrated by the team. The team discussed and agreed upon how data items would be selected and coded. In all, 2 authors (MS and LK) tested and calibrated self-developed data-charting forms for each study design until all relevant data were captured. The full data charting was conducted independently by 2 authors (MS and LK), and any discrepancies were discussed until consensus was reached.

### Data Items

For each article, the data extracted included the following:

1. Bibliographic information: title, first author, year of publication, and country.
2. Study and participant characteristics: study design, aim of the study, sample size, age, and gender of participants.
3. Characteristics of the digital intervention: mode or type, aim, and content.
4. Engagement measures: user engagement rate for the intervention, type of tool used to measure user engagement, and features of engagement measured.
5. Engagement strategies: strategies for user engagement used in the design and effective evaluated user engagement strategy.

Effective engagement strategies were identified for each article based on the authors' analysis of subjective user experience obtained through qualitative methodologies or questionnaires or, if available, based on the percentage of participants engaging with the intervention for a specific duration as determined by objective measures of intervention use.

### Study Quality

Consistent with the PRISMA-ScR [12] guidelines, Joanna Briggs Institute Scoping Review Methodology guidance, and the framework proposed by Arksey and O’Malley [14]**,** a quality appraisal was not conducted.

### Data Synthesis

The data were divided into groups based on study design. The outcomes were narratively synthesized for each study design.

## Results

### Selection of Sources of Evidence

**Figure 1:**
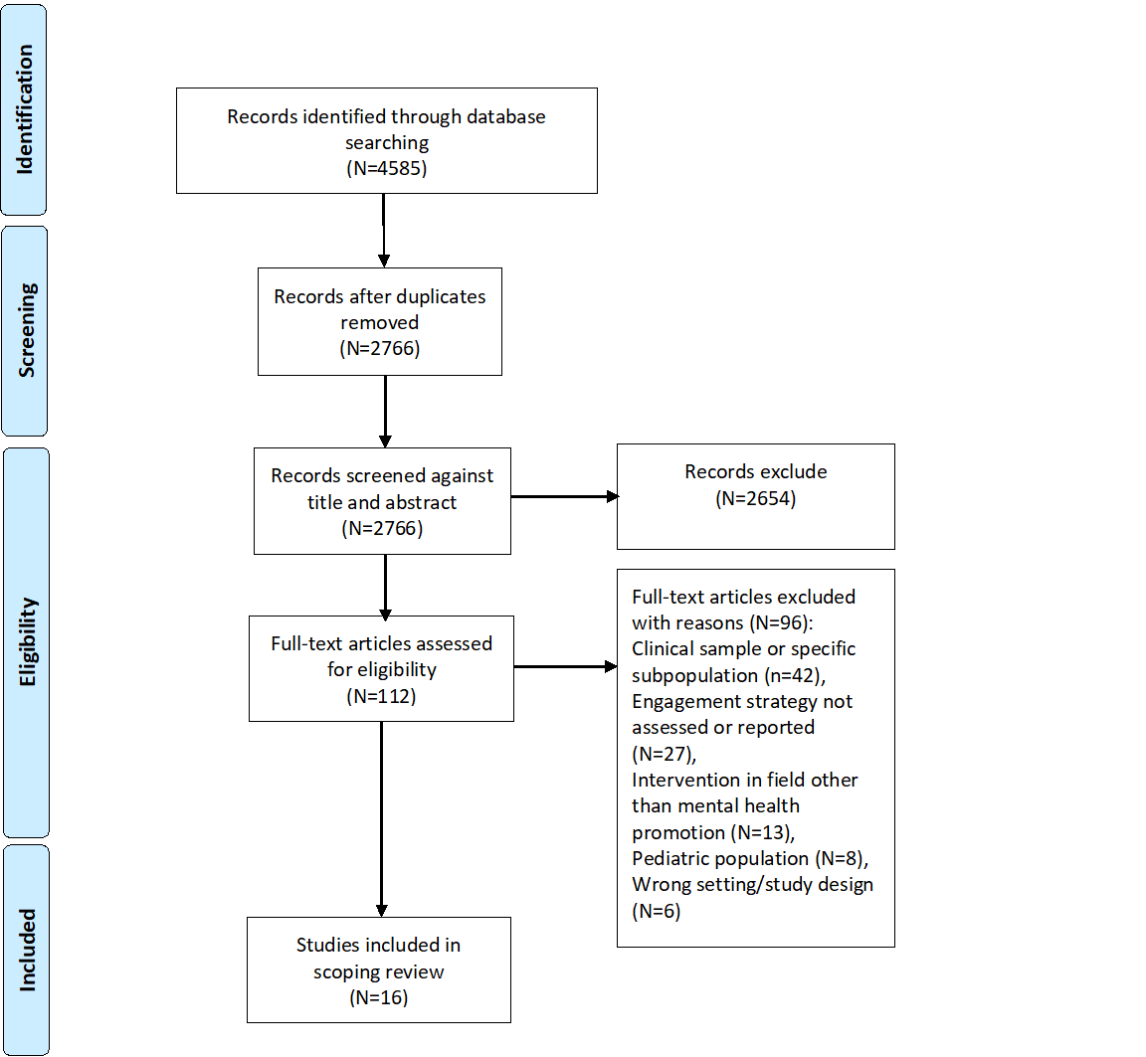
PRISMA (Preferred Reporting Items for Systematic Reviews and Meta-Analyses) flow diagram.

A total of 4585 articles were identified across all databases. Of the 4585 articles, 1819 (39.67%) duplicates were removed. On screening of the titles and abstracts of the remaining 2766 studies, 2654 (95.95%) were excluded, and the full texts of 112 (4.05%) articles were downloaded and screened against the inclusion and exclusion criteria. Of these 112 articles, 96 (85.7%) were excluded, and a total of 16 (14.3%) articles were included in this review. Figure 1 provides an overview of the selection process of the articles.

### Study Characteristics

Table 1 presents the general description of the studies. Of the 16 studies, 16 (100%) were published between 2013 and 2020 and originated from Europe (6/16, 37%), North America (6/16, 37%), and Australia (4/16, 25%). The study designs included randomized controlled trials (6/16, 37%), process data studies (5/16, 31%), observational studies (3/16, 19%), and qualitative studies (2/16, 13%). A process data study is a study conducting a secondary analysis on the primary data sets. The digital interventions for mental health promotion were mostly web based (12/16, 75%) or mobile app based (4/16, 25%).

**Table 1:** General characteristics of studies (N=16).

Author, year of publication; country^a^	Study design	Type of study	Intervention type (intervention name)
Lappalainen et al, 2013 [15]; Finland	RCT^b^	Feasibility	Web portal (P4 Well)
Todkill and Powell, 2013 [16]; United Kingdom	Qualitative study	Evaluation of design	Web based (MoodGym)
Morris et al, 2015 [17]; United States	RCT	Efficacy	Web based (Panoply)
Clarke et al, 2016 [18]; Australia	Process data study	Evaluation of usability engagement and efficacy	Web based (myCompass)
Laurie and Blandford, 2016 [19]; United Kingdom	Qualitative study	Gain insight into user experience	Mobile app (Headspace)
Zarski et al, 2016 [20]; Germany	Process data study	Evaluation of use in 3 intervention groups of RCTs	Web based and mobile app (GET.ON Stress)
Chou et al, 2017 [21]; United States	Process data study	Evaluation of design	Web based and mobile app (SuperBetter)
Dryman et al, 2017 [22]; United States	Process data study	Evaluation of use, engagement, and efficacy	Web based (Joyable)
Ly et al, 2017 [23]; Sweden	RCT	Feasibility	Mobile app (Shim)
Bakker et al, 2018 [24]; Australia	Cross-sectional	Evaluation of usability and feasibility	Mobile app (MoodMission)
Morrison et al, 2018 [25]; United Kingdom	Observational study	Efficacy	Web based and mobile app (Healthy Paths and Healthy Mind)
Song et al, 2018 [26]; Canada	Process data study	Evaluation of use and engagement	Web based (Walk Along)
Birk and Mandryk, 2019 [27]; United States	RCT	Efficacy	Web based (NR^c^)
Carter et al, 2019 [28]; United States	Cross-sectional	Evaluation of design	Mobile app (NewCope)
Przybylko et al, 2019 [29]; Australia	RCT	Efficacy	Web based (The Lift Project)
Renfrew et al, 2020 [30]; Australia	RCT	Comparison of 3 modes of engagement strategies	Web based and mobile app (MyWellness)

^a^Country of the corresponding author.

^b^RCT: randomized controlled trial.

^c^NR: not reported.

### Qualitative Studies

#### Overview

Of the 16 included studies, 2 (13%) were qualitative studies (36 participants). Both studies reported effective engagement strategies based on user feedback.

#### Engagement Strategies Used in the Design

Todkill and Powel [16], conducted a qualitative study with 20 participants who used the intervention for 12 weeks. The intervention included of a total of five modules that taught relaxation and meditation techniques, one module per week, web-based workbooks with 29 web-based exercises. No engagement strategy was reported.

Laurie and Blandford [19], conducted a qualitative study with 16 participants who used the intervention for 30 days. The intervention consisted of one audio file for daily guided meditation exercises for 10-15 minutes and a supplementary videos every 3-4 days. The researchers reported following engagement strategies used in intervention design (1) primary task support (audio and video content for meditation); (2) guidance (meditation guided by audio content); (3) third-party endorsement (during sign-up, users read quotes from journalists and celebrities); (4) social support (built-in buddy feature, allowing users to team up with others); (5) trust in provider.

#### Recommended Engagement Strategies

Both studies recommended the provision of daily challenging content and flexibility and ease of use as useful engagement strategies.

### Observational and Process Data Studies

#### Overview

Of the 16 included studies, 3 (19%) were observational studies (592 participants) and 5 (31%) were process data studies (7000 participants). Table 2 presents the findings of these studies.

**Table 2:** Overview of observational and process data studies (n=8).

Study	Number of participants	Intervention (duration)	Engagement strategy in intervention design	Recommended engagement strategy (author conclusion based on engagement rate)
Clarke et al, 2016 [18]	90	Assessment of users’ self-reported symptoms followed by 24×7 access to a personalized intervention that includes real-time self-monitoring of moods and interactive psychotherapeutic modules (7 weeks)	1. Reminders to facilitate self-monitoring by SMS text messaging or email as scheduled by the user; 2. Graphical feedback about self-monitoring	1. Personalized feedback incorporating program content; 2. Alerts and reminders; 3. Flexibility in agenda and use
Zarski et al, 2016 [20]	395	A total of 7 modules composed of psychoeducation and exercises for every module (4-7 weeks)	CG^a^: Received intervention; IG^b^1: Personalized written feedback from e-coach on the completed exercises and reminder by e-coach once in 7 days (content-focused guidance); IG2: Personalized feedback and adherence monitoring on demand of participants (adherence-focused guidance)	Content-focused guidance
Chou et al, 2017 [21]	—^c^	Gamified challenges in browser-based community forum; players can invite other players to browser-based community to form allies (4 weeks)	1. Gaming language; 2. Social forum; 3. Bright graphics	NR^d^
Dryman et al, 2017 [22]	3439	A total of 5 modules: learning through psychoeducation, core skill development by cognitive restructuring, 2 exposure modules, and final graduation module (12 weeks)	1. Coaches paired with users to provide feedback and support through weekly calls; 2. Coach-initiated and automated emails to encourage participation and progress	Guidance and support through coaching
Bakker et al, 2018 [24]	44	Assessment of user inputs on distress, followed by daily coping activities or games (30 days)	1. Games designed with real-time coping strategies; 2. Rewards for daily completed games; 3. Push notifications of incomplete games; 4. Bright graphics	NR
Morrison et al, 2018 [25]	543	Tools to improve awareness of participants’ thoughts or behaviors and support change in thinking patterns and behaviors (NR)	IG1: web based; IG2: mobile app; 1. Simple and reduced content; 2. Easy accessibility; 3. Push notifications for incomplete tool	IG2: 1. Simple and reduced content; 2. Easy accessibility; 3. Push notifications for incomplete tool
Song et al, 2018 [26]	3076	Self-help tools and a secure account with access to additional resources and links (NR)	NR	Personal email invitations to visit the site
Carter et al, 2019 [28]	5	Daily task, user-specific feedback, informational resources, self-assessment page, journal page (NR)	1. Self-monitoring tools for stress; 2. Goal setting with daily task; 3. User-specific feedback on stress level; 4. Reminders and progress summary	Task with user-specific feedback and self-monitoring

^a^CG: control group.

^b^IG: intervention group.

^c^Not available.

^c^NR: not reported.

#### Engagement Strategies Used in the Design

Of the 16 studies, 4 (25%) integrated personalized feedback about intervention content and users’ stress level as an engagement strategy in the intervention design and used reminder SMS text messaging or email according to users’ demand and progress as an engagement strategy. Moreover, of the 16 studies, 3 (19%) used bright colors and neat graphics as an engagement strategy in the intervention design. Guidance regarding content and progress through e-coaching was used in the intervention design of 13% (2/16) of the studies. Gamification of the content was used as an engagement strategy in the intervention design of 13% (2/16) of the studies. Goal setting and providing rewards were engagement strategies integrated into the intervention design of 13% (2/16) of the studies. Push notifications were identified in 13% (2/16) of the studies, whereas a social forum and interactivity with peers, simple content, and flexibility and ease of use were identified as the engagement strategies used in 6% (1/16) of the studies, separately.

#### Recommended Engagement Strategies

Effective engagement strategies were identified by the authors based on usability data and user feedback. The engagement measures of these studies are presented in Multimedia Appendix 5 [18,20-22,24-26,28]. Personalized feedback about intervention content and users’ stress level was identified as an effective engagement strategy in 50% (4/8) of the studies. For example, Clarke et al [18] reported on the myCompass program that assessed users’ self-reported symptoms on registration and provided personalized intervention with real-time self-monitoring of moods and behaviors and sent graphical feedback about users’ self-monitoring history alongside contextual information to their phone or PC as an engagement strategy and concluded that it directly enhanced users’ engagement with the program.

In 25% (2/8) of the studies, guidance regarding content and progress through e-coaching was reported as a strategy with the potential to increase engagement. In 25% (2/8) of the studies, implementing reminders according to users’ demand and progress was identified as a beneficial engagement strategy. For example, Zarski et al [19] analyzed 3 forms of guidance through human support and compared the effects on engagement with a stress-management intervention that involved content-focused guidance, adherence-focused guidance, and administrative guidance. Participants in the content-focused guidance group received personalized feedback after completion of every exercise. Participants in the adherence-focused guidance group received a personalized reminder by an e-coach in case of incomplete exercises. Participants in the administrative guidance group were provided with contact information during the study period. Participants in the content-focused guidance group showed the highest engagement, followed by participants in the adherence-focused guidance group and the sample that received administrative guidance. However, content-focused guidance was not significantly associated with higher adherence compared with adherence-focused guidance, indicating that guidance regarding content and progress through e-coaching improved engagement. Flexibility and ease of use was recommended in 25% (2/8) of the studies as an engaging strategy.

### Randomized Controlled Trials

#### Overview

Of the 16 included studies, 6 (37%) were experimental studies (15 arms). Table 3 presents the findings of these studies.

**Table 3:** Overview of randomized controlled trials (n=6).

Study	Participants, n (% female)	Age in years, mean (SD)	Intervention (duration)	Engagement strategy in intervention design by group	Recommended engagement strategy (author conclusion based on the usability of study arms)
Lappalainen et al, 2013 [15]	24 (0)	47 (7)	A total of 3 group meetings and personal use of web portal, apps, and devices (3 months)	CG^a^: No intervention; IG^b^: Personalized feedback	IG: Personalized feedback
Morris et al, 2015 [17]	166 (72)	24 (5)	Peer-to-peer platform for cognitive reappraisal and socioaffective support and supportive reappraisals from web-based crowd helpers (3 weeks)	CG: Expressive writing, web based; IG: 1. Short content; 2. Positive support messages from peers; 3. Quick feedback any time; 4. Repeated reminders; 5. Accessibility any time	IG: 1. Personalized feedback; 2. Support messages from peers; 3. Flexibility in use
Ly et al, 2017 [23]	28 (54)	26 (7)	Learn strategies of positive psychology through fully automated conversation, daily check-ins, and weekly summaries (2 weeks)	CG: No intervention; IG: Fully automated chatbot (conversational agent)	IG: Fully automated chatbot
Birk and Mandryk, 2019 [27]	259 (51)	35 (11)	Customization of avatar, ABMT^c^, and negative mood induction (3 weeks)	IG1: Customized avatar and ABMT; IG2: Customized avatar and no ABMT; IG3: Generic avatar and ABMT; IG4: Generic avatar and no ABMT	IG1: Avatar customization and personalization
Przybylko et al, 2019 [29]	426 (69)	47 (16)	Video presenting evidence-based strategies for promoting mental health and emotional wellness, daily and weekly challenges, gamification, and social forum (12 weeks)	CG: No intervention; IG: 1. Real-time coping strategies for daily mission; 2. Social forum with interactive component; 3. Rewards for completing missions; 4. Mission history available; 5. Push notifications alert for incomplete mission; 6. Bright graphics	IG: Interactive components in the social forum
Renfrew et al, 2020 [30]	458 (78)	46 (1)	Videos, workbook, reading materials related to the topic, and daily and weekly challenges (10 weeks)	CG: Automated email support; IG1: Automated email support and reminder SMS text messaging; IG2: Automated email support and videoconference session per week and 1 reminder SMS text message for videoconference per week	None (Videoconference mode had no effect on intervention engagement, and getting the chosen support style did not result in better engagement or outcomes)

^a^CG: control group.

^b^IG: intervention group.

^c^ABMT: attention bias modification training.

#### Engagement Strategies Used in the Design

In all, 4 intervention arms in 50% (3/6) of the studies used reminder SMS text messaging or email according to users’ demand and progress as an engagement strategy. Personalized feedback about intervention content and users’ stress level was used as an engagement strategy in 2 intervention arms in 33% (2/6) of the studies. A social forum and interactivity with peers was an engagement strategy in 2 intervention arms in 33% (2/6) of the studies. Easy accessibility and flexibility was used as an engagement strategy in 1 intervention arm. Personalization of content was used as an engagement strategy in 3 intervention arms in 33% (2/6) of the studies. Videoconference sessions with an e-coach were used as an engagement strategy in an intervention arm in 17% (1/6) of the studies. Push notifications were integrated as an engagement strategy in the intervention arm in 17% (1/6) of the studies. Gamification of content was used as an engagement strategy in an intervention arm in 17% (1/6) of the studies.

Renfrew et al [30] compared 3 modes of support differing in resource requirements on the effectiveness of the intervention, that is, automated emails, personalized SMS text messaging, and facilitated videoconference. Participants in the email group received a weekly email before every session with a 20- to 25-second video motivating users to engage. The personalized SMS text messaging group received an automated email and a personalized SMS text message, with the participant’s first name, to prompt engagement, and signature of a research team member, thrice weekly for the first 3 weeks and then twice weekly for the remaining 7 weeks. The reduction in messages was carried out with the perception that support has a threshold and a surfeit of messages may reduce engagement. The members of the videoconference group received automated email support and were invited to attend a videoconference session once a week. User engagement was not significantly different among the 3 groups, although notable variability in responses within the groups was indicated by a large SD.

#### Recommended Engagement Strategies

Engagement strategies were reported by authors based on usability data of different study arms. Engagement measures are presented in Multimedia Appendix 6 [15,17,23,27,29,30]. Of the 6 studies, 2 (33%) identified personalized feedback about intervention content and users’ stress level as an effective engagement strategy, and a social forum and interactivity with peers was identified as a useful engagement strategy in 2 (33%) studies. For example, Przybylko et al [29] included a social forum in the intervention design, where the participants could comment and post pictures regarding daily content. It was concluded that this strategy was highly engaging for users.

Of the 6 studies, 2 (33%) identified content personalization as an effective engagement strategy. For example, Ly et al [23] reported that content personalization through a fully automated chatbot intervention, Shim, which made users learn, reflect, and practice positive psychology through adequate responses and feedback to user’s statements, was found to be highly engaging for users. Birk and Mandryk [27] reported that avatar personalization for the intervention content greatly improved task-specific user engagement.

### Features of Engagement and Tools Used

**Figure 2:**
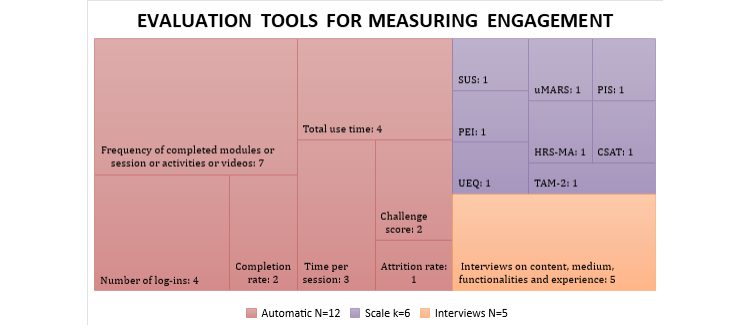
Evaluation tools for measuring engagement used in included studies. (Tool used for engagement measurement: number of studies using it). CSAT: Customer Satisfaction Scale; HRS-MA: Homework Rating Scale-Mobile Application; PEI: Patient Enablement Instrument; PIS: Player Identification Scale; SUS: System Usability Scale; TAM-2: Technology Acceptance Model-2; UEQ: User Experience Questionnaire; uMARS: Mobile Application Rating Scale, user version.

Table 4 and Figure 2 present the features of engagement explored in the different studies and the tools used to measure these features. Of the 16 studies, 13 (81%) measured frequency; 8 (50%) measured duration; 7 (44%) measured amount, attention, affect, and interest; and 3 (19%) measured depth. Of the 16 studies, 12 (75%) used automatic measures, 7 (44%) used self-administered questionnaires, and 5 (31%) used qualitative interviews to evaluate engagement.

**Table 4:** Features of engagement and measuring methods (N=16).

Study	Theory of engagement	Features of engagement	Tool used to measure engagement
Lappalainen et al, 2013 [15]	Technology tools	Affect	Questionnaire on perceived utility and acceptance
Lappalainen et al, 2013 [15]	Technology tools	Frequency	Automatic+questionnaire and number of log-ins
Todkill and Powell, 2013 [16]	—^a^	Affect, attention, and interest	Interviews on content, medium, functionalities, and experience
Morris et al, 2015 [17]	Technological interaction and consumption	Attention, interest, and depth	UEQ^b^+interview
Morris et al, 2015 [17]	Technological interaction and consumption	Amount, duration, and frequency	Automatic; time per session, time of intervention use total, and number of log-ins
Clarke et al, 2016 [18]	—	Affect	Interview
Clarke et al, 2016 [18]	—	Frequency	Automatic; number of log-ins, number of modules completed, frequency of self-monitoring, and interviews
Laurie and Blandford, 2016 [19]	—	Affect, attention, and interest	Interview and qualitative interviews
Laurie and Blandford, 2016 [19]	—	Frequency	Automatic; frequency of app use over study period
Zarski et al, 2016 [20]	—	Duration and frequency	Automatic; number of completed modules
Chou et al, 2017 [21]	—	Interest, depth, duration, and frequency	Automatic; completion rate of intervention
Dryman et al, 2017 [22]	Motivational techniques delivered by coaches	Frequency and duration	Automatic; average number of days in the program, average activities per participant, and completion rate of all modules
Ly et al, 2017 [23]	Fully automated conversational agent	Affect, attention, and interest	Interview
Ly et al, 2017 [23]	Fully automated conversational agent	Frequency	Automatic; number of reflections completed, number of active days, open app ratio, and interviews on content, medium, and functionalities
Bakker et al, 2018 [24]	Recommendations by Bakker et al [31] (2016)	Affect, attention, interest, depth, and frequency	Questionnaire, uMARS^c^, text-entry questions, and HRS-MA^d^
Morrison et al, 2018 [25]	Recommendation by Dennison et al [32] (2013)	Affect and attention	Questionnaire on satisfaction with the intervention, PEI^e^, and TAM-2^f^
Morrison et al, 2018 [25]	Recommendation by Dennison et al [32] (2013)	Duration and frequency	Automatic; total time of intervention use, time per log-in, and number of log-ins
Song et al, 2018 [26]	—	Interest, amount, duration, and frequency	Automatic; number of pages accessed per session, time per session, goal conversion rate, number of returning users, bounce rate, and number of pages accessed per session
Birk and Mandryk, 2019 [27]	Self-determination theory	Attention	Questionnaire and Player Identification Scale
Carter et al, 2019 [28]	Patient engagement framework	Frequency, attention, and interest	Automatic+questionnaire; percentage of task completion per user, average completion time of tasks, average CSAT^g^ scale score, Nielsen–Shneiderman heuristics, and SUS^h^
Przybylko et al, 2019 [29]	Experiential pedagogical framework	Duration	Automatic; attrition rate
Renfrew et al, 2020 [30]	SAM^i^	Duration Frequency	Automatic; total duration of videos viewed, number of videoconference sessions attended, and challenge score

^a^Not available.

^b^UEQ: User Experience Questionnaire.

^c^uMARS: Mobile Application Rating Scale, user version.

^d^HRS-MA: Homework Rating Scale-Mobile Application.

^e^PEI: Patient Enablement Instrument.

^f^TAM-2: Technology Acceptance Model-2.

^g^CSAT: Customer Satisfaction.

^h^SUS: System Usability Scale.

^i^SAM: Supportive Accountability Model.

## Discussion

### Principal Findings

This scoping review aims to identify the strategies that improve user engagement and explore how the engagement is evaluated in the context of digital interventions for mental health promotion. The findings from this scoping review suggest that there are 6 strategies that can positively influence engagement, with various design features to implement them. The methods to measure engagement included objective measures of technology use and subjective measures of user experience through questionnaires or qualitative interviews.

The key finding of our review is that strategies such as personalization, e-coaching, social forums, reminders, gamification, and flexibility and ease of use seem to promote engagement with digital interventions for mental health promotion.

### Comparison With Prior Work

Our review corresponds to previous findings in the broader literature of digital health well-being interventions and digital behavior change interventions, which concluded that personalization, support, and guidance through the intervention’s duration can increase user engagement and uptake [9,10]. Similar to previous studies, one of the most recommended strategies for increasing engagement identified in this review is e-coaching and human support [9,10,33]. Another interesting finding of this review is that personalization of intervention content or advanced design features that mimic human support, such as an automated chatbot or avatar customization, can increase engagement. This was also recently demonstrated in an experimental study on a smoking cessation app: users who were provided support through an automated chatbot were found to have higher engagement than users without the automated chatbot [34]. Other engagement strategies identified in this review include reminders, gamification (goals and rewards), and flexibility and ease of use. Likewise, Perski et al [10] identified reminders and incentives as engaging strategies in digital behavior change interventions. The authors also reported certain delivery features that they hypothesized to positively influence engagement. These included an esthetic design, ease of use, and the right message tone.

In general, digital interventions for mental health need to adopt some suitable strategies to motivate users to take up and continue use as well as use the full potential of the intervention [9]. In the following section, each of the identified engagement strategies and design features to tailor them are discussed in turn.

### Engagement Strategies

#### Personalization

Engagement strategies that incorporate personalization and allow customizing to user requirements and needs seem to enhance engagement [15-18,23,26-28]. The included studies used various design features to tailor personalization, including feedback on content, feedback on stress level, and personalization of intervention content. Personalized feedback and personalization of content were identified as strategies with the potential to increase user engagement.

The results here are comparable to those across mental health [35] and other areas of health promotion, such as in smoking cessation [36], physical activity promotion [37], and suicide prevention [38]. In the initial stage, the intervention can be tailored to user expectations on autonomy versus support. Accordingly, the level and kind of support provided during the intervention can be adapted to the user’s preference [5]. Examples of individualized support are personalized feedback and reminders [5].

#### e-Coaching

Guidance through e-coaching is another engagement strategy identified in this scoping review. The included studies [20,22,30] have used content-focused guidance and adherence-focused guidance design features, and evaluation has found them to greatly increase user engagement.

Previous research has demonstrated that e-coaching led to better engagement with digital interventions for mental health. Persuasive e-coaching and guidance have been associated with better treatment outcome, engagement, and retention in psychological web-based interventions for the treatment of depression according to a systematic review [33]. Although a recent scoping review concluded that providing structured support improved engagement with an internet-based psychological intervention, the variability in the provision of human support, such as delivery mode, intensity, and type, resulted in heterogeneous outcomes, making comparisons difficult [39].

#### Social Forum

Social forums and interactivity with peers has been identified as a strategy to increase user engagement with digital interventions for mental health promotion [17,21,29]. This has also been supported by recent studies that found that human interaction of any kind is greatly valued by users of digital interventions for mental health [6]. Therefore, human influence should be accorded the same priority as the technology itself [1]. A narrative review recommended that social forums and social media should be harnessed to provide mental health services for youth to increase access to, and engagement with, digital therapeutic solutions for their mental health [40].

#### Reminders

Reminders have been identified as an engagement strategy in various included studies [17,18,22,25,28-30]. Different design features have been used to tailor reminders: push notifications, personalized SMS text messaging, personalized email, reminder SMS text messaging or email by an e-coach according to the use pattern of the user, and passive reminder SMS text message or email. Personalized SMS text messaging and reminder SMS text messaging or email by an e-coach according to the use pattern of the user have the potential to greatly increase engagement.

Consistent with this finding, a factorial screening experiment explored the impact of 4 different types of SMS text messages on a behavior change smoking cessation intervention and demonstrated that reminders through SMS text messaging based on users’ use pattern of content can boost overall levels of engagement with the intervention [41].

#### Gamification

Gamification of content has been identified as an engagement strategy in this scoping review [19,21,24,28,29]. Various design features have been used in the intervention designs of the included studies to tailor gamification. These include gamification of content, goal setting, rewards or badges for a completed mission, and provision of new content daily.

A systematic review examining the effect of gamification on adherence to web-based interventions for mental health treatment concluded that various gamification features have been incorporated in the design of web-based interventions. The effect of gamification on user engagement and adherence remains inconclusive because this has not been explored explicitly [42].

#### Flexibility and Ease of Use

Flexibility and ease of use was identified as an engagement strategy in this review. Various design features such as flexibility of content use, offline availability of content, bright graphics, big colorful icons, easy-to-understand content, and web-based and mobile app options have been used in the included studies to tailor this strategy. In line with this, the latest literature review analyzed users’ public reviews for mental health apps to gain insights into user perceptions and concluded that ease of use was a feature both liked and recommended by users of mental health apps [35].

### Methods to Evaluate Engagement

The methods to evaluate engagement in the included studies can be broadly described as objective users’ use measures and subjective experience measures. The result demonstrates heterogeneous reporting of engagement measures and a wide range of assessment measures and reporting data. The heterogeneity of engagement data makes the result incomparable and hinders the understanding of the effectiveness of engagement strategies. Consistent with this finding, other reviews examining engagement with health and well-being apps also reported heterogeneity of data and incomparable results [9]. In addition, the reviews examining the effectiveness of different design features of a single engagement strategy reported inconclusive findings because of the heterogeneity of data [39,42].

Similarly, a recent systematic review investigating measurement and reporting methods of user engagement with mental health apps concluded that high heterogeneity of the measuring and reporting methods and different methodologies used to assess mental health apps, such as user satisfaction, acceptability, feasibility, and usability, make it difficult to report actual engagement with these apps. In addition, there is a need for careful understanding of engagement before claiming engagement strategies used by these apps as effective engagement strategies [43].

### Recommendation for Future Design and Research

First, the engagement strategies identified and recommended in this scoping review were primarily explored as a by-product in the included studies and were not evaluated systematically. Of the 16 studies, only 2 (13%) [20,30] were identified that methodologically explored the effectiveness of different engagement strategies for user engagement. The remaining studies merely recommended strategies based on the authors’ analysis of user feedback or participant use data for the intervention. This shows that, so far, the focus has only been on effectiveness, acceptability, feasibility, or use of digital technologies for mental health promotion and there is a lack of interest in the effectiveness of engagement strategies because the interventions address healthy people rather than clinical samples. Thus, more experimental studies are required to investigate the effectiveness of engagement strategies for digital technologies for mental health promotion based on the percentage of participants who report their engagement with such technologies.

In addition, the identified engagement strategies are presented as a separate component in this scoping review. However, these strategies have been used in combination in the intervention designs of the included studies. A review of digital mental health interventions recommended the incorporation of different persuasive technological features that can result in different synergies compared with their use [44]. Therefore, future studies should focus on exploring and evaluating various engagement strategies, their dosage, and different combinations to identify the most effective set of strategies for use and engagement.

Second, engagement was explored and measured heterogeneously. Even among studies with the same designs, the reported engagement data were heterogeneous, making it impossible to determine the most effective engagement strategy. The approach to implementing engagement strategies for digital health interventions is relatively new and highly varied. There is a need for harmonization of research, evaluation, and reporting standards to produce high-quality evidence for engagement. This could be achieved with the development and adoption of guidelines or a minimum set of indicators to measure engagement. Furthermore, digital technologies allow for the creation of large data sets that may be used to assess outcomes based on engagement with specific intervention components [45]. More research is required to identify what characteristics or correlates of engagement can be consistently recorded over time to investigate how engagement changes over time and how engagement is related to the intervention’s duration, as well as to develop new statistical approaches for analyzing these large and complex data sets. Future research should also report attrition rates to explore possible relationships between engagement and attrition.

Third, research is required on the *healthy level* of engagement to achieve the desired outcomes and reduce attrition. In general, the optimal *dose* of engagement is still unclear in the field of digital health interventions. Yardley et al [46] propose that establishing and promoting “effective engagement” rather than merely “more engagement” may be more useful for digital interventions for behavior change, with “effective engagement” defined as “sufficient participation with the intervention to accomplish desired effects.” The findings of Zhang et al [47] suggest that digital apps addressing mental health should follow the Goldilocks concept of *just right*. Like many other digital technologies, mental health apps do not ensure that *the more the engagement, the better the results*. The benefits of using an app can only be achieved when the dosages of various sorts of intervention features are *just right*. Exhaustion can result from too much engagement, whereas inefficiency might result from too little engagement. As a result, mental health technology should be designed in such a way that it encourages optimal use [47]. Fourth, engagement was not explored in depth. Most of the studies explored the objective measures of technology use, including frequency, duration, and amount, whereas subjective measures of user experience, such as affect, attention, and interest, were explored less commonly. Engagement with digital interventions for behavior change can be described as the extent of use and subjective experience [10]. Perski et al [10] conceptualized engagement with digital behavior change interventions and proposed a framework through a systematic interdisciplinary approach to assess different features of engagement. The authors emphasized that the future research avenue should include assessment of all features of engagement to reduce the fragmentation in digital health research and establish standard optimal procedures to achieve engagement across different kinds of digital behavior change interventions [10]. Although objective measures of technology use can provide data on user engagement, the exploration of subjective measures of user experience can help in identifying correlates of engagement. Therefore, future studies should explore engagement features in depth, that is, both objective use and subjective experience measures, to generate better evidence for engagement with digital interventions.

### Limitations

To our knowledge, this is the first scoping review to explore engagement strategies for digital interventions for mental health promotion. The findings have been summarized taking into consideration the authors’ recommendations based on user engagement data or user feedback. Nevertheless, this review includes a few limitations. It focuses on digital interventions for mental health promotion for the nonclinical population; thus, the findings may not be applicable to other settings. Another limitation is that our inclusion criteria were very narrow for a scoping review, and it cannot be ruled out that studies with clinical samples included healthy control groups that could have been included in this scoping review. This review included only published data and excluded gray literature; therefore, some relevant literature may have been missed.

### Conclusions

Various engagement strategies have been reported in digital interventions for mental health promotion, including personalization, human and social support, gamification, personalized feedback, and reminders. User engagement was predominantly reported in terms of frequency, duration, and amount, as well as subjectively (affect, attention, and interest). Human support and e-coaching during the intervention, access to social support, human support–mimicking design features, and personalized feedback or reminders may work best to promote engagement. The findings need to be interpreted with caution because the included studies were heterogeneous, had small sample sizes, and typically explored engagement strategies only as a by-product. All studies were from high-income, nonclinical settings that may not be applicable to other contexts. Despite the importance of user engagement for the effectiveness of digital interventions, this area has not yet received much attention; therefore, conclusions cannot be drawn regarding the most effective engagement strategy because of the heterogeneity of data. Further experimental research is needed on the effectiveness of different types of engagement strategies to facilitate user engagement with digital interventions for mental health promotion.

## Appendix

Multimedia Appendix 1 PRISMA (Preferred Reporting Items for Systematic Reviews and Meta-Analyses) Extension for Scoping Reviews checklist.

Multimedia Appendix 2 Systematic searches of electronic databases.

Multimedia Appendix 3 Search syntax for primary studies in MEDLINE.

Multimedia Appendix 4 Full-text assessment and list of excluded studies.

Multimedia Appendix 5 Overview of observational and process data studies.

Multimedia Appendix 6 Overview of experimental studies.

## Abbreviations

DALY: disability-adjusted life year
PRISMA-ScR: Preferred Reporting Items for Systematic Reviews and Meta-Analyses Extension for Scoping Reviews
