# Supplementary material for: Understanding Engagement Strategies in Digital Interventions for Mental Health Promotion: Scoping Review
Source: JMIR Ment Health. 2021 Dec 20;8(12):e30000. doi: 10.2196/30000 (PMC8726056; doi:10.2196/30000)
Supplement: Multimedia Appendix 2 [file mental_v8i12e30000_app2.pdf]

Multimedia Appendix File 2. Systematic searches of electronic databases

Database	Platform	Timeframe	Search Result	Deduplicates Search Result
Medline	Ovid	(1946–April 2020)	1262	1244
Cumulative Index to Nursing and Allied Health Literature (CINAHL)	Ebsco	(1981–April 2020)	1100	490
The Social Science Citation Index (SSCI)	Web of Science	(1900–April 2020)	597	140
Science Citation Index (SCI)		(1900–April 2020)		
Emerging Sources Citation Index (ESCI)		(2015–April 2020)		
PsycInfo	Ovid	(1806–April 2020)	690	417
CENTRAL and the Cochrane Database of Systematic Reviews (CDSR)	Cochrane Library	(inception to April 2020)	936	477
