# Supplementary material for: Understanding Engagement Strategies in Digital Interventions for Mental Health Promotion: Scoping Review
Source: JMIR Ment Health. 2021 Dec 20;8(12):e30000. doi: 10.2196/30000 (PMC8726056; doi:10.2196/30000)
Supplement: Multimedia Appendix 3 [file mental_v8i12e30000_app3.pdf]

Multimedia Appendix File 3. Search syntax for primary studies in Medline

Line	*Search*
1	((digital or mobile or ehealth or "e health" or mhealth or "m health" or online or web* or internet or computer* or technolog* or app? or automatic* or automate?) adj5 intervention*).mp.
2	(("cognitive behavio?r* therap*" or CBT) adj5 (mobile or electronic* or internet or web or online or computer* or digital* or technolog* or app?)).mp.
3	digital behavio?r* change intervention*.mp.
4	icbt*.mp.
5	"behavio?r* change technique*".mp.
6	exp mobile applications/
7	exp telemedicine/
8	exp "internet-based intervention"/
9	or/1-8
10	((patient? or user? or consumer? or client? or participant? or people or individual?) adj5 (incentive* or reminder* or attrition or usage or tailoring or engag* or adher* or interact* or prompt* or adopt* or participat* or retention or retain* or feedback or interact*)).mp.
11	exp patient dropouts/
12	exp patient compliance/
13	or/10-12
14	((emotion* or psycholog* or mental) adj3 (health or stress* or problem* or disturb* or aspect* or state* or ill or illness or disorder* or disease* or wellbeing or well-being or wellness)).mp.
15	(("mental health" or depression* or dysthymi* or melancholi* or stress* or anxiet* or "emotional health" or depressive* or depressed or anxious) adj5 (prevention* or preventative or promotion* or initiative* or campaign*)).mp.
16	exp mental health/
17	exp depression/
18	exp anxiety/
19	exp stress, psychological/
20	exp health promotion/
21	or/14-20
22	and/9,13,21
