# Supplementary material for: Understanding Engagement Strategies in Digital Interventions for Mental Health Promotion: Scoping Review
Source: JMIR Ment Health. 2021 Dec 20;8(12):e30000. doi: 10.2196/30000 (PMC8726056; doi:10.2196/30000)
Supplement: Multimedia Appendix 4 [file mental_v8i12e30000_app4.pdf]

Multimedia Appendix File 4. Full-text assessment and list of excluded studies

**List of excluded studies (N=96)**
Reason: Clinical sample or specific subpopulation (N=42)
[1–42]
Reason: Engagement strategy not assessed/reported (N=28)
[43–70]
Reason: Intervention in other field than mental health promotion (N=13)
[71–83]
Reason: Paediatric population (N=8)
[84–91]
Reason: Wrong setting/study design (N=6)
[92–96]

References:

[1] Abd-Alrazaq AA, Alajlani M, Alalwan AA, et al. An overview of the features of chatbots in mental health: A scoping review. *International Journal of Medical Informatics* 2019; 132: 103978.

[2] Alvarez-Jimenez M, Gleeson JF, Bendall S, et al. Enhancing social functioning in young people at Ultra High Risk (UHR) for psychosis: A pilot study of a novel strengths and mindfulness-based online social therapy. *Schizophrenia Research* 2018; 202: 369–377.

[3] Armstrong L, Rimes KA. Mindfulness-Based Cognitive Therapy for Neuroticism (Stress Vulnerability): a Pilot Randomized Study. *Behavior Therapy* 2016; 47: 287-298.

[4] Batterham PJ, Han J, Mackinnon AJ, et al. Factors associated with engagement in online self-help programs among people with suicidal thoughts. *Journal of Affective Disorders* 2020; 265: 402–409.

[5] Bennett M, Harris N, Learmonth D, et al. Banishing the blues. *Mental Health Practice* 2008; 12: 32–36.

[6] Bolier L, Ketelaar SM, Nieuwenhuijsen K, et al. Workplace mental health promotion online to enhance well-being of nurses and allied health professionals: a cluster-randomized controlled trial. *Internet Interventions* 2014; 1: 196-204.

[7] Carolan S, Harris PR, Cavanagh K. Improving Employee Well-Being and Effectiveness: Systematic Review and Meta-Analysis of Web-Based Psychological Interventions Delivered in the Workplace. *Journal of Medical Internet Research* 2017; 19: e271.

[8] Christie GI, Shepherd M, Merry SN, et al. Gamifying CBT to deliver emotional health treatment to young people on smartphones. *Internet Interventions* 2019; 18: 100286.

[9] Isrctn. Evaluating the effectiveness of "Agoraphobia Free": a novel mobile application for treating Agoraphobia. *http://www.who.int/trialsearch/Trial2.aspx?TrialID=ISRCTN98453199*.

[10] Clarke G, Eubanks D, Kelleher C, et al. Overcoming Depression on the Internet (ODIN) (2): A Randomized Trial of a Self-Help Depression Skills Program With Reminders. *Journal of Medical Internet Research* 2005; 7: No Pagination Specified.

[11] Crisp DA, Griffiths KM. Reducing Depression Through an Online Intervention: Benefits From a User Perspective. *JMIR Mental Health* 2016; 3: e4.

[12] Crisp DA, Griffiths KM. Participating in online mental health interventions: who is most likely to sign up and why? *Depression Research and Treatment* 2014; 2014: 790457.

[13] Danaher BG, Milgrom J, Seeley JR, et al. Web-Based Intervention for Postpartum Depression: Formative Research and Design of the MomMoodBooster Program. *JMIR Research Protocols* 2012; 1: e18.

[14] Ebert DD, Buntrock C, Lehr D, et al. Effectiveness of Web- and Mobile-Based Treatment of Subthreshold Depression With Adherence-Focused Guidance: A Single-Blind Randomized Controlled Trial. *Behavior Therapy* 2018; 49: 71–83.

[15] Fleischmann RJ, Harrer M, Zarski AC, et al. Patients' experiences in a guided Internet- and App-based stress intervention for college students: a qualitative study. *Internet Interventions* 2018; 12: 130-140.

[16] Gibson K, Trnka S. Young people's priorities for support on social media: "it takes trust to talk about these issues." *Computers in Human Behavior* 2020; 102: 238–247.

[17] Grime PR. Computerized cognitive behavioural therapy at work: a randomized controlled trial in employees with recent stress-related absenteeism. *Occupational Medicine (Oxford)* 2004; 54: 353–359.

[18] Heilemann MS V., Martinez A, Soderlund PD. A mental health storytelling intervention using transmedia to engage latinas: Grounded theory analysis of participants' perceptions of the story's main character. *Journal of Medical Internet Research* 2018; 20: 1.

[19] Inal Y, Wake JD, Guribye F, et al. Usability Evaluations of Mobile Mental Health Technologies: Systematic Review. *Journal of Medical Internet Research* 2020; 22: e15337.

[20] Isrctn. Using online-cognitive behaviour therapy (Online-CBT) to treat general anxiety and worry among older adults: is it effective and does client engagement matter? *http://www.who.int/trialsearch/Trial2.aspx?TrialID=ISRCTN83626400*.

[21] Karyotaki E, Kleiboer A, Smit F, et al. Predictors of treatment dropout in self-guided web-based interventions for depression: An "individual patient data" meta-analysis. *Psychological Medicine* 2015; 45: 2717–2726.

[22] Kelders SM, Bohlmeijer ET, Pots WT, et al. Comparing human and automated support for depression: Fractional factorial randomized controlled trial. *Behaviour Research & Therapy* 2015; 72: 72–80.

[23] Kelders SM, Bohlmeijer ET, Van Gemert-Pijnen JE. Participants, usage, and use patterns of a web-based intervention for the prevention of depression within a randomized controlled trial. *Journal of Medical Internet Research* 2013; 15: e172.

[24] Khan S. The role of linguistic agency, message causality and screen size in increasing engagement with mental health video games app. *Dissertation Abstracts International Section A: Humanities and Social Sciences* 2019; 80: No Pagination Specified.

[25] Kubo A, Kurtovich E, McGinnis M, et al. A Randomized Controlled Trial of mHealth Mindfulness Intervention for Cancer Patients and Informal Cancer Caregivers: A Feasibility Study Within an Integrated Health Care Delivery System. *Integrative Cancer Therapies* 2019; 18: 1534735419850634.
[26] Linardon J, Fuller-Tyszkiewicz M. Attrition and adherence in smartphone-delivered interventions for mental health problems: A systematic and meta-analytic review. *Journal of Consulting & Clinical Psychology* 2020; 88: 1–13.
[27] Lipschitz J, Miller CJ, Hogan TP, et al. Adoption of Mobile Apps for Depression and Anxiety: Cross-Sectional Survey Study on Patient Interest and Barriers to Engagement. *JMIR Mental Health* 2019; 6: e11334.
[28] McCall HC, Helgadottir FD, Menzies RG, et al. Evaluating a Web-Based Social Anxiety Intervention Among Community Users: Analysis of Real-World Data. *Journal of Medical Internet Research* 2019; 21: e11566.
[29] Mehrotra S, Tripathi R. Recent developments in the use of smartphone interventions for mental health. *Current Opinion in Psychiatry* 2018; 31: 379–388.
[30] Morgan C, Mason E, Newby JM, et al. The effectiveness of unguided internet cognitive behavioural therapy for mixed anxiety and depression. *Internet Interventions-the Application of Information Technology in Mental and Behavioural Health* 2017; 10: 47–53.
[31] Morrison C, Walker G, Ruggeri K, et al. An implementation pilot of the MindBalance web-based intervention for depression in three IAPT services. *Cognitive Behaviour Therapist*; 7. Epub ahead of print 2014. DOI: 10.1017/S1754470X14000221.
[32] Morthland MP. Technology assisted intervention for improving mood: A portable computer-assisted therapy program for treating depression in older adults. *Dissertation Abstracts International: Section B: The Sciences and Engineering* 2012; 73: 1261.
[33] Nitsch M, Dimopoulos CN, Flaschberger E, et al. A Guided Online and Mobile Self-Help Program for Individuals With Eating Disorders: An Iterative Engagement and Usability Study. *Journal of Medical Internet Research* 2016; 18: e7.
[34] Owen JE, Jaworski BK, Kuhn E, et al. mHealth in the Wild: Using Novel Data to Examine the Reach, Use, and Impact of PTSD Coach. *JMIR Mental Health* 2015; 2: e7.
[35] Pratap A, Renn BN, Volponi J, et al. Using Mobile Apps to Assess and Treat Depression in Hispanic and Latino Populations: Fully Remote Randomized Clinical Trial. *Journal of Medical Internet Research* 2018; 20: e10130.
[36] Rodriguez MA. The use of task-sharing to improve treatment engagement in an online mindfulness intervention for stress among Chinese college students.

Dissertation Abstracts International: Section B: The Sciences and Engineering 2019; 80: No Pagination Specified.
[37] Sanatkar S, Baldwin PA, Huckvale K, et al. Using Cluster Analysis to Explore Engagement and e-Attainment as Emergent Behavior in Electronic Mental Health. *Journal of Medical Internet Research* 2019; 21: e14728.
[38] Simblett S, Matcham F, Siddi S, et al. Barriers to and Facilitators of Engagement With mHealth Technology for Remote Measurement and Management of Depression: Qualitative Analysis. *JMIR MHealth and UHealth* 2019; 7: e11325.
[39] Takahashi K, Takada K, Hirao K. Feasibility and preliminary efficacy of a smartphone application intervention for subthreshold depression. *Early intervention in psychiatry* 2019; 13: 133–136.
[40] Whitton AE, Proudfoot J, Clarke J, et al. Breaking Open the Black Box: Isolating the Most Potent Features of a Web and Mobile Phone-Based Intervention for Depression, Anxiety, and Stress. *JMIR Mental Health* 2015; 2: e3.
[41] Zarski AC, Berking M, Reis D, et al. Turning Good Intentions Into Actions by Using the Health Action Process Approach to Predict Adherence to Internet-Based Depression Prevention: Secondary Analysis of a Randomized Controlled Trial. *Journal of Medical Internet Research* 2018; 20: e9.
[42] Actrn. Behavioural and Cognitive Changes During Self-Guided Internet-Delivered Cognitive-Behavioural Therapy for Anxiety and Depression: a Randomised Controlled Trial. *http://www.who.int/trialsearch/Trial2.aspx?TrialID=ACTRN12615000112561*.
[43] Aboujaoude E, Gega L. From Digital Mental Health Interventions to Digital “Addiction”: Where the Two Fields Converge. *Frontiers in psychiatry Frontiers Research Foundation* 2019; 10: 1017.
[44] Barbabella F, Poli A, Andreasson F, et al. A Web-Based Psychosocial Intervention for Family Caregivers of Older People: Results from a Mixed-Methods Study in Three European Countries. *JMIR Research Protocols* 2016; 5: e196.
[45] Batterham PJ, Calear AL, O’Dea B, et al. Stakeholder perspectives on evidence for digital mental health interventions: Implications for accreditation systems. *Digital Health* 2019; 5: 2055207619878069.
[46] Beintner I, Vollert B, Zarski AC, et al. Adherence Reporting in Randomized Controlled Trials Examining Manualized Multisession Online Interventions: Systematic Review of Practices and Proposal for Reporting Standards. *Journal of Medical Internet Research* 2019; 21: e14181.
[47] Isrctn. Perinatal psychological care to mothers and fathers of SGA infants. *http://www.who.int/trialsearch/Trial2.aspx?TrialID=ISRCTN15627704*.

[48] Christensen H, Reynolds J, Griffiths KM. The use of e-health applications for anxiety and depression in young people: Challenges and solutions. *Early intervention in psychiatry* 2011; 5: 58–62.

[49] de Beurs D, van Bruinessen I, Noordman J, et al. Active Involvement of End Users When Developing Web-Based Mental Health Interventions. *Frontiers in psychiatry Frontiers Research Foundation* 2017; 8: 72.

[50] Actrn. HeadGear – A mental health smartphone application for the prevention of depression: a randomised controlled trial. *http://www.who.int/trialsearch/Trial2.aspx?TrialID=ACTRN12617000548336*.

[51] Duggleby W, Ploeg J, McAiney C, et al. A Comparison of Users and Nonusers of a Web-Based Intervention for Carers of Older Persons With Alzheimer Disease and Related Dementias: mixed Methods Secondary Analysis. *Journal of Medical Internet Research* 2019; 21: e14254-.

[52] Ervasti M, Kallio J, Maattanen I, et al. Influence of Personality and Differences in Stress Processing Among Finnish Students on Interest to Use a Mobile Stress Management App: Survey Study. *JMIR Mental Health* 2019; 6: e10039.

[53] Fitzsimmons-Craft EE, Firebaugh ML, Graham AK, et al. State-Wide University Implementation of an Online Platform for Eating Disorders Screening and Intervention. *Psychological Services* 2019; 16: 239–249.

[54] Fleming T, Bavin L, Lucassen M, et al. Beyond the trial: Systematic review of real-world uptake and engagement with digital self-help interventions for depression, low mood, or anxiety. *Journal of Medical Internet Research* 2018; 20: 1–11.

[55] Forbes L, Gutierrez D, Johnson SK. Investigating Adherence to an Online Introductory Mindfulness Program. *Mindfulness* 2018; 9: 271–282.

[56] Graham AK, Lattie EG, Mohr DC. Experimental Therapeutics for Digital Mental Health. *JAMA Psychiatry* 2019; 76: 1223–1224.

[57] Kaltenthaler E, Cavanagh K, McCrone P. Evaluating the role of electronic and web-based (E-CBT) CBT in mental health. *Technology in mental health: Applications in practice, supervision and training , 2nd ed* 2016; 212–222.

[58] Kinderman P, Hagan P, King S, et al. The feasibility and effectiveness of Catch It, an innovative CBT smartphone app. *BJPsych Open* 2016; 2: 204–209.

[59] King K, Schlichthorst M, Turnure J, et al. Evaluating the effectiveness of a website about masculinity and suicide to prompt help-seeking. *Health Promotion Journal of Australia* 2019; 30: 381–389.

[60] Lal S, Adair CE. E-mental health: a rapid review of the literature. *Psychiatric Services* 2014; 65: 24–32.

[61] Lara MA, Tiburcio M, Aguilar Abrego A, et al. A four-year experience with a Web-based self-help intervention for depressive symptoms in Mexico. *Pan American Journal of Public Health* 2014; 35: 399–406.

[62] Lara MA, Tiburcio Sainz M. Dropout in self-guided web-based interventions for depression can be predicted by several variables. *Evidence-Based Mental Health* 2016; 19: e3.
[63] Lattie EG, Adkins EC, Winquist N, et al. Digital mental health interventions for depression, anxiety and enhancement of psychological well-being among college students: Systematic review. *Journal of Medical Internet Research* 2019; 21: e12869.
[64] Lee EW, Denison FC, Hor K, et al. Web-based interventions for prevention and treatment of perinatal mood disorders: A systematic review. *BMC Pregnancy and Childbirth* 2016; 16: 1–8.
[65] Moberg C, Niles A, Beermann D. Guided Self-Help Works: Randomized Waitlist Controlled Trial of Pacifica, a Mobile App Integrating Cognitive Behavioral Therapy and Mindfulness for Stress, Anxiety, and Depression. *Journal of Medical Internet Research* 2019; 21: e12556.
[66] Nygren T, Berg M, Sarkohi A, et al. Development of an Internet-Based Cognitive Behavioral Therapy Self-Help Program for Arabic-Speaking Immigrants: Mixed-Methods Study. *JMIR Research Protocols* 2018; 7: e11872.
[67] Proudfoot J, Clarke J, Birch MR, et al. Impact of a mobile phone and web program on symptom and functional outcomes for people with mild-to-moderate depression, anxiety and stress: a randomised controlled trial. *BMC Psychiatry* 2013; 13: 312.
[68] Schueller SM, Glover AC, Rufa AK, et al. A Mobile Phone-Based Intervention to Improve Mental Health Among Homeless Young Adults: Pilot Feasibility Trial. *JMIR MHealth and UHealth* 2019; 7: e12347.
[69] van Straten A, Cuijpers P, Smits N. Effectiveness of a web-based self-help intervention for symptoms of depression, anxiety, and stress: randomized controlled trial. *Journal of Medical Internet Research* 2008; 10: e7.
[70] Chittaro L, Vianello A. Evaluation of a mobile mindfulness app distributed through on-line stores: A 4-week study. *International Journal of Human Computer Studies* 2016; 86: 63–80.
[71] Burger F, Neerincx MA, Brinkman WP. Technological state of the art of electronic mental health interventions for major depressive disorder: Systematic literature review. *Journal of Medical Internet Research* 2020; 22: 1–22.
[72] Cavanagh K. Turn on, tune in and (don't) drop out: Engagement, adherence, attrition, and alliance with internet-based interventions. *Oxford guide to low intensity CBT interventions* 2010; 227–233.
[73] Chen AT, Wu S, Tomasino KN, et al. A multi-faceted approach to characterizing user behavior and experience in a digital mental health intervention. *Journal of Biomedical Informatics* 2019; 94: 103187.

[74] Clough BA, Eigeland JA, Madden IR, et al. Development of the eTAP: A brief measure of attitudes and process in e-interventions for mental health. *Internet Interventions* 2019; 18: 100256.
[75] Gulliver A, Bennett K, Bennett A, et al. Privacy Issues in the Development of a Virtual Mental Health Clinic for University Students: A Qualitative Study. *JMIR Mental Health* 2015; 2: e9.
[76] Hanisch SE, Birner UW, Oberhauser C, et al. Development and Evaluation of Digital Game-Based Training for Managers to Promote Employee Mental Health and Reduce Mental Illness Stigma at Work: Quasi-Experimental Study of Program Effectiveness. *JMIR Mental Health* 2017; 4: e31.
[77] Hobson GR, Caffery LJ, Neuhaus M, et al. Mobile Health for First Nations Populations: Systematic Review. *JMIR MHealth and UHealth* 2019; 7: e14877.
[78] Musiat P, Goldstone P, Tarrier N. Understanding the acceptability of e-mental health--attitudes and expectations towards computerised self-help treatments for mental health problems. *BMC Psychiatry* 2014; 14: 109.
[79] Nicholas J, Fogarty AS, Boydell K, et al. The Reviews Are in: A Qualitative Content Analysis of Consumer Perspectives on Apps for Bipolar Disorder. *Journal of Medical Internet Research* 2017; 19: e105.
[80] Omar A, Wahlqvist ML, Kouris-Blazos A, et al. Wellness management through Web-based programmes. *Journal of Telemedicine and Telecare* 2005; 11: 8–11.
[81] Schueller SM, Neary M, O'Loughlin K, et al. Discovery of and Interest in Health Apps Among Those With Mental Health Needs: Survey and Focus Group Study. *Journal of Medical Internet Research* 2018; 20: e10141.
[82] Actrn. OL@-OR@ trial: a cluster-randomised controlled trial to evaluate a co-designed, culturally-tailored, lifestyle-support mobile health (mHealth) tool, which includes an app and website for Maori and Pasifika in New Zealand. *http://www.who.int/trialsearch/Trial2.aspx?TrialID=ACTRN12617001484336*.
[83] Actrn. Doctors Working Well: a Study Evaluating an Online Stress Management Program for Doctors. *http://www.who.int/trialsearch/Trial2.aspx?TrialID=ACTRN12617000290392*.
[84] Cheng VWS, Davenport TA, Johnson D, et al. An App That Incorporates Gamification, Mini-Games, and Social Connection to Improve Men's Mental Health and Well-Being (MindMax): Participatory Design Process. *JMIR Mental Health* 2018; 5: e11068.
[85] Dubad M, Winsper C, Meyer C, et al. A systematic review of the psychometric properties, usability and clinical impacts of mobile mood-monitoring applications in young people. *Psychological Medicine* 2018; 48: 208–228.
[86] Garrido S, Millington C, Cheers D, et al. What Works and What Doesn't Work? A Systematic Review of Digital Mental Health Interventions for Depression and

Anxiety in Young People. *Frontiers in psychiatry Frontiers Research Foundation* 2019; 10: 759.
[87] Martínez-Miranda J, Martínez A, Ramos R, et al. Assessment of users' acceptability of a mobile-based embodied conversational agent for the prevention and detection of suicidal behaviour. *Journal of Medical Systems* 2019; 43: N.PAG-N.PAG.
[88] Orlowski SK, Lawn S, Venning A, et al. Participatory Research as One Piece of the Puzzle: A Systematic Review of Consumer Involvement in Design of Technology-Based Youth Mental Health and Well-Being Interventions. *JMIR Human Factors* 2015; 2: e12.
[89] Shandley K, Klein B, Austin D. The players' perspective of Reach Out Central: A therapeutic interactive online game. *E-Journal of Applied Psychology* 2008; 4: 51–55.
[90] Windler C, Clair M, Long C, et al. Role of Moderators on Engagement of Adolescents With Depression or Anxiety in a Social Media Intervention: Content Analysis of Web-Based Interactions. *JMIR Mental Health* 2019; 6: e13467.
[91] Actrn. A Randomised Controlled Trial Investigating Online Cognitive Behavioural Therapy for Perfectionism to Prevent Eating Disorders. *http://www.who.int/trialsearch/Trial2.aspx?TrialID=ACTRN12615000128594*.
[92] Carpenter J, Crutchley P, Zilca RD, et al. "Seeing the 'big' picture: Big data methods for exploring relationships between usage, language, and outcome in internet intervention data": Correction. *Journal of Medical Internet Research Vol 19(12), 2017, ArtID e347*; 19. Epub ahead of print 2017. DOI: http://dx.doi.org/10.2196/jmir.8099.
[93] Khazaal Y. [Mental Health apps: innovations and challenges]. *Revue Medicale Suisse* 2019; 15: 1650–1656.
[94] Lattie EG, Graham AK, Hadjistavropoulos HD, et al. Guidance on defining the scope and development of text-based coaching protocols for digital mental health interventions. *Digital Health* 2019; 5: 2055207619896145.
[95] Musiat P, Hoffmann L, Schmidt U. Personalised computerised feedback in E-mental health. *Journal of Mental Health* 2012; 21: 346–354.
[96] Torous J, Nicholas J, Larsen ME, et al. Clinical review of user engagement with mental health smartphone apps: evidence, theory and improvements. *Evidence-Based Mental Health* 2018; 21: 116–119.
