# Supplementary material for: Understanding Engagement Strategies in Digital Interventions for Mental Health Promotion: Scoping Review
Source: JMIR Ment Health. 2021 Dec 20;8(12):e30000. doi: 10.2196/30000 (PMC8726056; doi:10.2196/30000)
Supplement: Multimedia Appendix 5 [file mental_v8i12e30000_app5.pdf]

Multimedia Appendix File 5: Overview of observational and process data studies

Author, year of publication	Number of participants[c]	Intervention description and duration	Engagement strategy in intervention design	Effective engagement strategy according to authors' conclusion	Engagement rates
Clarke et al [18], 2016	90	Intervention assesses users' self-reported symptoms and then provides 24/7 access to a personalized intervention that includes real-time, self-monitoring of mood, interactive psychotherapeutic modules (7 weeks)	1. Reminders to facilitate self-monitoring by SMS or email as scheduled by the user<br>2. Graphical feedback about self-monitoring	1. Personalized feedback incorporating program content<br>2. Alerts and reminders<br>3. Flexibility in agenda and usage	NR
Zarski et al [20], 2016	395	7 modules composed of psychoeducation and exercises for every module (4 to 7 weeks)	CG: Intervention	IG1: Content focus guidance	CG: M=4.4 (SD 2.8)<br>(Mean (M) number of completed treatment modules)
			IG1: Personalized written feedback from e-coach on the completed exercises + reminder by e-coach once in 7 days (content-focused guidance)		IG1: M=5.7 (SD 2.3)
			IG2: Personalized feedback and adherence monitoring on demand of participants (adherence-focused guidance)		IG2: M=5.6 (SD 2.3)
Chou et al [21], 2017	NR	Gamified challenges in browser-based community forum Players can invite other players to browser-based community. To form allies (4 weeks)	1. Gaming language<br>2. Social forum<br>3. Brightly colored layout; aesthetically pleasing icons	NR	Completion rate: 20%<br>(Completion rate: percentage of users that completed 4-weeks intervention)
Dryman et al [22], 2017	3439	Five modules: learning through psychoeducation, core skill development by cognitive restructuring, Two exposure modules, and final graduation module (12 weeks)	1. Coaches paired with users to provide feedback and support through weekly calls<br>2. Coach-initiated and automated emails to encourage participation and progress	1. Guidance and support through coaching	Average activities/participant: 12<br>All module completion rate:16%<br>(Average activities/ participant: average

					of total number of activities completed by all participants<br><u>All module completion rate:</u> percentage of participants completing all 5 modules)
Bakker et al [24] 2018	44	Intervention assesses user inputs on distress and provides with daily coping activities/games (30 days)	1.Games designed with real-time coping strategies.<br>2. Rewards for daily completed games<br>2. Push notifications of incomplete games<br>3. Clean graphic design	NR	NR
Morrison et al [25], 2018	543	Tools to improve awareness of their thoughts or behaviors and support change in thinking patterns and behaviors (NR)	IG1: (web-based)	IG2:<br>1. Simple and reduced content<br>2. Easy Accessibility<br>3. Push notifications for incomplete tool	NR
			IG2: (mobile app)<br>1. Simple and reduced content<br>2. Easy Accessibility<br>3. Push notifications for incomplete tool		NR
Song et al [26], 2018	3076	Self-help tools, a secure account with access to additional resources and links (NR)	NR	Personal email invitations to visit the site	Session rate: 2420 (45.51%).<br>Bounce rate 36.6%,<br>N of pages/ session:7.6,<br>Mean session duration: 3 min 42 s,<br>Conversion rate: 24.4%<br>(<u>Session rate:</u><br>The percentage of new visits.<br><u>Bounce rate:</u> Bounce rate is single-page

					sessions divided by all sessions.<br>N of Pages/ session: dividing the<br>total number of page views by the total number of sessions<br>Conversion rate:<br>Number of goal conversions divided by the number of sessions)
Carter et al [28], 2019	5	Daily task, user-specific feedback, Informational resources, self-assessment page, journal page (NR)	1. Self-monitoring tools for stress<br>2. Goal setting with daily task<br>3. User-specific feedback on stress level<br>4. Reminders and progress summary	Task with user-specific feedback and self-monitoring	Task completion rate: 40-80%<br>Task completion rate: Each task completed by participant divided total number of participants

Note: N: Number, NR: not reported. M: mean, SD: standard deviation. IG: Intervention group, CG: Control group
