# Supplementary material for: Understanding Engagement Strategies in Digital Interventions for Mental Health Promotion: Scoping Review
Source: JMIR Ment Health. 2021 Dec 20;8(12):e30000. doi: 10.2196/30000 (PMC8726056; doi:10.2196/30000)
Supplement: Multimedia Appendix 6 [file mental_v8i12e30000_app6.pdf]

Multimedia Appendix File 6: Overview of experimental studies

Study	N (%female)<br>Age M±SD	Intervention (duration)	Engagement strategy in intervention design by group	Effective engagement strategy (author conclusion based on the usability of study arms)	Engagement rates of different study arms
Lappalainen et al [15], 2013	24 (0%)<br>47 ± 7	3 group meetings + personal use of web portal, apps, and devices (3 months)	CG: No intervention	IG: Personalized feedback	NR
			IG: Personalized feedback		
Morris et al [17], 2015	166 (72%)<br>24±5	Peers to peer's platform for cognitive reappraisal and socio-affective support, supportive reappraisals from online crowd helpers (3 weeks)	CG: Expressive writing web-based	1. Personalized feedback<br>2. Support messages from peers<br>3. Flexibility in usage	CG: 75.2% completed follow-up
			IG:<br>1. Short content<br>2. Positive support messages from peers<br>3. Quick feedback anytime<br>4. Repeated reminders<br>5. Accessibility anytime		IG: 77.7% completed follow-up
Ly et al [23], 2017	28<br>(54%)<br>26±7	Learn strategies of positive psychology through fully automated conversation, daily check-ins, weekly summaries (2 weeks)	CG: No intervention	IG: Fully automated chatbot	
			IG: Fully automated chatbot (conversational agent)		IG: 13/14 completed intervention (defined as at least 14 reflections over the course of 14 days, as well as not inactive for 7 or more days in a row)
	259 (51%)<br>35±11	Customization of avatar, Attention Bias	IG1: Customized avatar/ABMT		NR

Birk and Mandryk [27], 2019		Modification Training (ABMT), negative mood induction<br>(3 weeks)	IG2: Customized avatar/ no ABMT	IG1: Avatar customization and personalization	
			IG3: Generic avatar/ABMT		
			G4: Generic avatar/no ABMT		
Przybylko et al [29], 2019	426 (69%)<br>47±16	A video that presented evidence-based strategies for promoting mental health and emotional wellness, daily and weekly challenges, gamification, Social forum<br>(12 weeks)	CG: No intervention	IG: Interactive components in the social forum	
			IG:<br>1. Real-time coping strategies for daily mission,<br>2. Social forum with interactive component<br>3. Rewards for completing missions.<br>4. Mission history available<br>5. Push notifications alert for incomplete mission<br>6. Clean graphic design reduces confusion.		IG; Attrition Rate: 24%
Renfrew et al [30], 2020	458 (78%)<br>46±1	Videos, workbook, reading materials relating to the topic, daily and weekly challenges (10 weeks)	CG: Automated email support	IG2: Videoconference session + SMS reminders	CG: Mv=6.05 (SD 4.0)<br>CG: Mc=369 (SD 362)<br>CG: AR= 21.9%<br>(Mean (Mv) Number of videos watched out of 10.<br>Mean (Mc) challenge adherence score out of 1000.<br>Attrition Rate (AR) total number of randomized participants who did not complete either the preintervention (ie, early dropout) or the postintervention (ie, lost to follow-up) questionnaire.)

			IG1: Automated email support + reminder SMS		IG1: Mv=6.48 (SD 3.9)<br>IG1: Mc=340 (SD 339)<br>IG1: AR=19.3%
			IG2: Automated email support + videoconference session/ week + 1 reminder SMS for videoconference/ week		IG2: Mv=6.75 (SD 3.8)<br>IG2: Mc=377 (SD 354)<br>IG2: AR=31.6%

Note: N: Number, NR: not reported. M: mean, SD: standard deviation. IG: Intervention group, CG: Control group
